# Microbiology and immune mechanisms associated with male infertility

**DOI:** 10.3389/fimmu.2023.1139450

**Published:** 2023-02-21

**Authors:** Jin Chen, Jinyu Chen, Yiwei Fang, Qiuzi Shen, Kai Zhao, Chunyan Liu, Huiping Zhang

**Affiliations:** ^1^ Institute of Reproductive Health, Tongji Medical College, Huazhong University of Science and Technology, Wuhan, China; ^2^ Department of Urology, Union Hospital, Tongji Medical College, Huazhong University of Science and Technology, Wuhan, China

**Keywords:** microbiology, immune, infertility, varicocele, orchitis, prostatitis, oligospermia

## Abstract

Up to 50% of infertility is caused by the male side. Varicocele, orchitis, prostatitis, oligospermia, asthenospermia, and azoospermia are common causes of impaired male reproductive function and male infertility. In recent years, more and more studies have shown that microorganisms play an increasingly important role in the occurrence of these diseases. This review will discuss the microbiological changes associated with male infertility from the perspective of etiology, and how microorganisms affect the normal function of the male reproductive system through immune mechanisms. Linking male infertility with microbiome and immunomics can help us recognize the immune response under different disease states, providing more targeted immune target therapy for these diseases, and even the possibility of combined immunotherapy and microbial therapy for male infertility.

## 1 Introduction

Infertility has been defined as the inability to conceive after at least 12 months of regular and unprotected sexual intercourse by WHO (1). It has brought substantial economic and psychological burden to the society (2). Nearly 15% of couples are affected by infertility, with male infertility contributing 50% among them (3). A review published in Lancet summarized some common factors that may cause male infertility, including varicocele, orchitis, prostatitis, oligospermia, asthenospermia, azoospermia and other male diseases (4).

The human microbiome is an ecosystem composed of many kinds of microorganisms. It is a relatively balanced state, not absolutely sterile, and mainly exists in external cavities such as the reproductive tract, oral cavity, and gastrointestinal tract. External bacteria, viruses, fungi, mycoplasma, chlamydia infection or opportunistic bacterial infection will cause the occurrence and development of a variety of diseases, male reproductive-diseases are also included (5). At present, more and more studies have been published on the relationship between microorganisms and male infertility. Male infertility caused by microbial infection is mostly achieved through immune response, and microorganisms induce the accumulation of immune cells and proinflammatory cytokines and chemokines. And the production of anti-sperm antibodies and biofilms also can damage germ cells and destroy the normal spermatogenic function (6).

At present, there is no systematic review on the immune mechanisms of several common diseases of male infertility (varicocele, orchitis, prostatitis, oligozoospermia, asthenospermia, azoospermia) caused by microbial infections. This review will fill this gap, aiming to provide new ideas and targets for the treatment of male infertility.

## 2 Varicocele

### 2.1 Varicocele and microbiology

Varicocele is an important cause of male infertility. A large number of studies have shown that varicocele causes spermatogenesis and sperm dysfunction through oxidative stress. As for whether there are microbial colonization changes in the semen of patients with varicocele, recent studies have shown that the colonization of Ureaplasma urealyticum in patients with varicocele is significantly higher than that in healthy men. However, there is no direct evidence of the damage of Ureaplasma urealyticum to sperm, the oxidative stress caused by Ureaplasma urealyticum may have an adverse effect on semen quality (7). It is also possible that the co-infection of Chlamydia trachomatis, Ureaplasma urealyticum, and Mycoplasma hominis with the inflammation of the varicocele may lead to the loss of seminal ocilium, resulting in male infertility (8). Toxoplasma patients complicated with varicocele also can lead to abnormal sperm (9). In addition, varicocele may be associated with viral infection, but the correlation may be weak and more studies still needed to be explored. HSV was detected in 7% of 14 varicocele patients at one time, while CMV was detected in only two of 29 infertile men with varicocele in another study (10). Vicari et al. found that HBV and HCV were associated with varicocele. The sperm-related parameters of infertile men with HBV and varicocele were significantly lower than those of infertile men with only varicocele or HCV combined with varicocele. Therefore, HBV may aggravate the sperm damage of patients with varicocele (11).

### 2.2 The immune mechanism in the varicocele microbiology

#### 2.2.1 Immune cells and cytokines

Bacteria and viruses can interact with the host’s immune and inflammatory systems through their unique structure to elevate the polymorphonuclear leucocytes and granulocytes, resulting in oxidative stress and impaired male fertility (12). Recent studies have found that even though the concentration of white blood cells is normal in VC patients, the subsets of white blood cells are significantly changed, the proportion of CD8+ T cells and macrophages is significantly reduced, while the level of CD4+ Th cells is significantly increased, leading to the increase of cytokine levels in VC patients (13). Due to the increase of oxidative stress level in VC patients, it can mediate the change of immune cells in testis, further lead to irreversible damage of testis and affect normal spermatogenesis (14).

By binding to. specific receptors on the surface of target cells, cytokines control the intracellular response and expression of related genes, the details are shown in **Table 1**. In the earlier study of Adel et al., it was observed that the concentration of IL-6 was significantly increased in cases of varicocele without inflammation, but it was not found to be significant in cases of varicocele with inflammation, so IL-6 was negatively correlated with sperm concentration and sperm motility (26). Later, Nallella et al. found a significant increase in IL-6 in patients with varicocele (22). Sakamoto et al. also got the similar result, they found that the IL-6 level in the varicocele group was significantly higher than that in the control group (27). In recent years, it has been further found that TNF-α impairs testis by changing mitochondrial function, increasing NO production and promoting oxidative stress. A recent study by Moretti et al. showed a positive correlation between IL-6 levels and the level of malondialdehyde (MDA) in semen. The levels of malondialdehyde(MDA) in seminal plasma and semen were negatively correlated with sperm parameters, which further confirmed that IL-6 was involved in oxidative stress induced sperm damage (28). IL-1α and IL-1β are two forms of IL-1, which are regulators of testicular function, but increased expression of IL-1α and IL-1β in rats with varicocelectic veins, especially in the 11 - and 13-week-old groups, disrupted the balance of inflammation and immunity and had deleterious effects on testicular tissue (15). Moreover, IL-37 and IL-18 were also found to be upregulated in the seminal plasma of varicocele patients (15). These elevations lead to inflammatory response activation, leucocyte recruitment and ROS production, which are detrimental to normal testicular functions (29). Importantly, ROS can disrupt the blood–testis barrier, the sperm plasma membrane and DNA integrity and then affected male fertility (30). The production of NO and inflammatory factors can also promote the assembly of NLRP3 inflammasome complex through the differentiation of testis macrophages, and the increase of inflammasome can in turn promote the production of ROS and affect the normal spermatogenic function of testis (31). In addition, studies have demonstrated that down-regulation of inflammasome can reduce the apoptosis of spermogenesis related cells in testicular tissue of VC patients (32).

**Table 1:** Cytokines and varicocele.

Cytokine	Species	Group	Sample Type	Regulation	Reference
IL-1α	Rats	VC & Sham	Testicular Tissue	Up	(15)
IL-1β	Rats	VC & Sham	Testicular Tissue	Up	(15)
IL-6	Rats	VC & Sham	Testicular Tissue	Up	(16)
IL-6	Rats	VC & Sham	Serum	Up	(16)
IFN-γ	Rats	VC & Sham	Testicular Tissue	Up	(16)
IFN-γ	Rats	VC & Sham	Serum	Up	(16)
IFN-γ	Rats	VC & Sham	Serum	Up	(16)
IL-18	Homo Sapiens	Infertility with VC & Normal	Seminal Fluid	Up	(17)
IL-37	Homo Sapiens	Infertility with VC & Normal	Seminal Fluid	Up	(17)
IL-6	Homo Sapiens	Infertility with VC & Normal	Seminal Fluid	Up	(18)
TNF-α	Homo Sapiens	Infertility with VC & Normal	Seminal Fluid	Up	(18)
IL-8	Homo Sapiens	Infertility with VC & Normal	Seminal Fluid	Up	(19)
IL-8	Homo Sapiens	Infertility with VC & Normal	Seminal Fluid	Up	(20)
IL-1β	Homo Sapiens	Infertility with VC & Normal	Seminal Fluid	Up	(21)
IL-6	Homo Sapiens	Infertility with VC & Normal	Seminal Fluid	Up	(22)
TNF-α	Homo Sapiens	Infertility with VC & Normal	Seminal Fluid	NSS	(23)
IFN-γ	Homo Sapiens	Infertility with VC & Normal	Seminal Fluid	Down	(24)
TGF-β	Rats	Infertility with VC & Normal	Testicular Tissue	Up	(25)

VC, varicocele; Up, Up-regulation of cytokine levels; Down, Down-regulation of cytokine levels; NSS, No statistical significance.

#### 2.2.2 Anti-sperm antibody

Another important immunological factor contributing to infertility in patients with varicocele is the presence of anti-sperm antibodies (ASAs). ASAs are found in 5%-15% of men with infertility (33). Animal experiments showed that the ASAs level of VC model rats was higher than that of normal rats (25). Similarly, studies in the population have further validated that. In varicocele patients, the autoimmune anti-sperm reaction is accompanied by a more significant decrease in the semen quality (33). ASAs can cause sperm agglutination and reduce sperm motility, resulting in male infertility, and even affect early implantation and pregnancy. In an earlier study, Golomb et al. observed that ASA was significantly present in the serum and seminal plasma of infertile men with varicocele, mainly IgA and IgM (34). Bozhedomov et al. showed an association between the grade of varicose veins and ASA level by direct current cytometry. Among ASA-positive patients, the more severe the varicose veins, the lower the sperm concentration, sperm density and sperm motility (33). For patients with varicose veins undergoing venous ligation, Djaladat et al. obtained that varicose vein surgery reduces ASA levels in some patients, but may also increase them in some patients, and this positive conversion does not adversely affect sperm parameters (35). A recent study found that the number of patients with active sperm autoimmunity was 2.8 times lower than that before surgery, and the average MAR-IgG level was 1.8 times lower in the group with improvement after surgery, but the presence of ASA reduced the efficacy of reproductive function recovery after varicocular surgery (36).

The pathogen interacts with the immune system of VC patients, resulting in increased levels of polymorphonuclear leucocytes, granulocytes and CD4+ Th, further increased levels of cytokines and inflammasome, and increased oxidative stress (12, 13). It changes the immune microenvironment in testis and affects male normal fertility. IL-6 is involved in the process of oxidative stress induced sperm damage, and can be considered as a biomarker (22, 26, 27). It is also very important to study the treatment of VC patients with ASAs, which affects the subsequent recovery of reproductive function (33–36).

## 3 Orchitis

### 3.1 Orchitis and microbiology

In the early stage of SARS-induced orchitis, extensive destruction of germ cells, few or no sperm in seminiferous tubules, thickening of basement membranes and infiltration of white blood cells are shown, affecting male reproductive function (37). The recent epidemic of COVID-19 has a high degree of sequence similarity with SARS. Studies in respiratory system have shown that COVID-19 invades organs through ACE2 receptors, the testicular Ledig and Sertoli cells highly express ACE2 receptors, so that virus can also achieve its invasion into the testis through ACE2 receptors (38). The increase of immune cells in the testicular interstitium and proinflammatory cytokines IL-6, TNF-α and MCP-1 in the semen of patients with COVID-19 reduces the concentration of sperm and impairs spermatogenesis. The accumulation of inflammatory cells and their products caused by the virus can activate an autoimmune response leading to autoimmune orchitis, which damages spermatogenic epithelium. In addition, persistent fever may also be the cause of germ cell damage and degeneration in COVID-19 patients (39).

Mumps virus often causes patient orchitis and affects male fertility function. Mumps orchitis usually occurs about a week after the onset of mumps. It may start with systemic symptoms and later manifest as swelling and pain in the testicles. MuV induces immune response with TLR2 and RIG-I signaling, resulting in an increase in proinflammatory cytokines and chemokines (40). MuV can also inhibit antiviral IFN signaling through AXL and MER receptors, thereby promoting excess MuV replication in Leydig and Sertoli cells, and may even induce anti-sperm antibodies with potentially long-term adverse effects on patient fertility (6).

Chlamydia is also often considered as an important pathogen of orchitis through the route of sexual transmission, of which Chlamydia trachomatis and Neisseria gonorrhoeae are the most common. During the period of Chlamydial infection, testicular cells can be observed that DNA damage and the transcription of epigenetic mediated disorder lead to abnormal sperm epigenome, moreover also can increase the leukocyte infiltration, destroy the blood-testis barrier, reduce the number of sperm cells and the volume of seminiferous tubule, leading to low fertility and birth defects (41).

### 3.2 The immune mechanism in the orchitis microbiology

#### 3.2.1 Immune cells

During the onset of autoimmune orchitis, the quantification and phenotype of infiltrating cells in the testis showed an increase in the number of T cell subsets, dendritic cells, and macrophages, including both Th and Treg cells. However, the active effect of Th cells exceeded that of accumulated Treg cells, and the presence of inflammation limited the ability of Treg cells to eliminate tissue damage. Treg cells usually prevent the induction of spontaneous organ-specific autoimmunity by persistent endogenous danger signals (42). Both CD4+ and CD8+ T cells increase in the initial inflammatory process, while CD4+ T cells decrease and CD8+ T numbers remain unchanged during the chronic maintenance of EAO. Therefore, CD4+ and CD8+ T cells play an advantage in the onset and chronic phase of EAO, respectively (43). For regulatory T cells, both CD4+Foxp3+ and CD8+Foxp3+ Treg cells were increased, and CD25+ cells were more common (43). In terms of another classification of T cells, both αβ and γδT cell subsets are increased in orchitis. αβT cells are the initiators of the autoaggressive response, and γδT cells play a regulatory role in infection-induced autoimmune orchitis (44).

Jing et al. found that cDC1 is necessary for the presence of T cells in the testis, but cDC2 does not have any significance for the maintenance of T cells, the depletion of T cells does not affect normal spermatogenesis, and cDC1-dependent T cells play an important role in chronic autoimmune orchitis (45).

Testicular macrophages are the major immune cells within the mammalian testis and are important for organogenesis, spermatogenesis, and androgen production, providing protection to the developing male germ cells while also allowing adequate response to inflammatory stimuli to produce proinflammatory immunity and anti-infection protection against invading pathogens. The ED1+ macrophage subsets are the main pathogenic subsets in the occurrence of EAO. HMGB1 can stimulate macrophages and phosphorylate p38 mitogen-activated protein kinase (MPPK) and p65 NF- κB, leading to increased TNF-α and IL-6 and causing testicular injury (46). While S100A9 by activating PI3K/Akt pathway to maintain the immunosuppressive function of macrophages (47). The same effect was observed in the macrophages of male genital tract infection caused by E. coli, which could not only increase the anti-inflammatory cytokines regulated by NFAT (calcium dependent nuclear factor), but also down-regulate the expression of proinflammatory factors in peritoneal macrophages (PM) inhibited by α-hemolysin. By LPS treated TM was not sensitive to the activation of NF- κB, and the secretion of proinflammatory factors was deficient. By this mechanism TM initiates an anti-infective response while maintaining testicular immune privilege and protecting spermatogenic cells (48).

#### 3.2.2 Cytokines and chemokines

Cytokines play an important role in the pathogenesis of orchitis. As shown in **Table 2**, some of the cytokines have changed in the orchitis model. Multiple studies have demonstrated the significant role of IL-6 in orchitis (50, 53, 54). Studies show that immune cells affect the normal immunosuppressive microenvironment of the testis by secreting cytokines such as TNF-α, IL-6, IL-12, IL-17 and IL-23, and proinflammatory cytokines can also play an indirect role by affecting the immunosuppressive effect of Treg cells (42). The same phenomenon was also observed in the study of Nicolas et al., who found that the expression of TNF, MCP-1, and IL-10 also increased as the increase of T cells (58). A large number of data on acute orchitis show that the up-regulation of IL-1β, IL-α, IL-6 and TNF-α can adversely affect germ cells, increasing germ cell expression and promoting germ cell apoptosis through the TNF-α/TNFR1, IL-6/IL-6R and Fas/FasL systems (59). Studies have shown that uropathogenic Escherichia Coli (UPEC) orchitis induces the activation of NLRP3 inflammasome through up-regulation of CaSR, promotes the secretion and maturation of IL-1β, and affects the synthesis of testosterone (56). IL-17A can promote the focal inflammatory cell infiltration in the testicular interstitium, resulting in the increased permeability of the blood-testis barrier and the loss of germ cells in the adjacent seminiferous tubules, thus affecting the spermatogenic function of the testis (52). In addition, high doses of IL-18 from immune cells *in vivo* can induce apoptosis of Leydig cells through the Fas pathway (60). The increase in chemokines such as CCL2, CCL3 and CCL4 can also be observed in EAO, inducing the attraction and extravasation of immune cells (59).Guazzone et al. also showed that the highest levels of CCL3 in the testis coincided with the onset of the disease (61).

**Table 2:** Cytokines and orchitis.

Cytokine	Species	Group	Sample Type	Regulation	Reference
IL-1β	Rats	EAO&Normal	Testicular Tissue	Up	(49)
IL-6	Rats	EAO&Normal	Testicular Tissue	Up	(50)
IL-6	Rats	EAO&Normal	Testicular Tissue	Up	(46)
TNF-α	Rats	EAO&Normal	Testicular Tissue	Up	(46)
TNF-α	Rats	EAO&Normal	Testicular Tissue	Up	(51)
IL-17A	Rats	EAO&Normal	Testicular Tissue	Up	(52)
IL-17A	Mice	EAO&Normal	Testicular Tissue	Up	(53)
IL-6	Mice	EAO&Normal	Testicular Tissue	Up	(53)
IFN-γ	Mice	EAO&Normal	Testicular Tissue	Up	(53)
TNF-α	Mice	EAO&Normal	Testicular Tissue	Up	(53)
IL-1β	Mice	EAO&Normal	Testicular Tissue	NSS	(53)
IL-6	Mice	EAO&Normal	Testicular Tissue	Up	(54)
TNF-α	Mice	EAO&Normal	Testicular Tissue	Up	(54)
IL-1β	Mice	UPEC&Normal	Testicular Tissue	Up	(55)
IL-1β	Rats	UPEC&Normal	Testicular Tissue	Up	(56)
IL-1β	Rats	UPEC&Normal	Testicular Tissue	Up	(57)
IL-6	Mice	UPEC&Normal	Testicular Tissue	Up	(55)
TNF-α	Mice	UPEC&Normal	Testicular Tissue	Up	(55)

NSS, No statistical significance; EAO, Experimental Autoimmune Orchitis; UPEC, Uropathogenic Escherichia Coli-induced orchitis; Up, Up-regulation of cytokine levels.

Based on our data and the other aforementioned studies, it is reasonable to propose that Th1 cells are augmented at the onset of orchitis, inducing anti-infection responses. However, in the chronic phase, Th17 cells dominate the Th cell subsets and maintain the inflammation state in the testes by inhibiting Tregs and crowding out other effector T cells. Collectively, Th cells in orchitis are involved in the impairment of the structure and spermatogenesis of the testes (58). Immune cells in the testis affect the normal immunosuppressive microenvironment of the testis by secreting cytokines and chemokines. The TNF-α/TNFR1, IL-6/IL-6R and Fas/FasL systems promote the apoptosis of germ cells (42, 59).

## 4 Prostatitis

### 4.1 Prostatitis and microbiology

#### 4.1.1 Bacteria

According to the internationally recognized National Institutes of Health (NIH) classification, there are five types of prostatitis: acute prostatitis type I, chronic bacterial prostatitis type II, chronic prostatitis/chronic pelvic pain syndrome type III (CP/CPPS), and asymptomatic prostatitis type IV. Chronic pelvic pain syndrome type II is the most common type, accounting for 90-95% of all prostatitis diagnoses. The prevalence of the disease in the general population is 5-14.2%, and it is more common in people aged 35-45 years (62, 63).

The most common infections for acute bacterial prostatitis are coliforms and enterococci, which are similar to other common urogenital infections. Most of them can be cured in the acute phase, and fewer of them will develop non-chronic prostatitis, which is more complicated. Infection with viruses, fungi, mycoplasma and chlamydia may also be involved.

Corynebacterium was found in the urine and semen of both chronic prostatitis patients and healthy men, but it was evident that in prostatitis patients with severe leukoospermia, Corynebacterium was found in more species and in higher concentration, and group G Corynebacterium and Arthrobacter were more representative, which has a higher correlation with inflammatory prostatitis (64). P. acnes, a common skin microorganism, is also found in patients with prostatitis. It induces the secretion of cytokines and chemokines, and causes inflammation of the prostate and affecting sperm function through the host protein vimentin expressed in the prostate for bacterial erosion (65).

Patients with CP/CPPS had higher levels of Clostridium and Burkholderia in their urine microbiota (66, 67). In addition, the differences between CP/CPPS patients and controls were also different according to the time of urine collection. Nickel et al. found significant differences in overall species and genus composition in the initial urine flow, whereas no significant differences were observed at any level in urine samples from the middle and late stages of prostate massage. Therefore, the results of urinalysis are specific and time-sensitive, and do not represent the overall level (67). Recent studies on CP/CPPS showed that Achromobacter, Oligotrophomonas, and Brevibacterium were more common, and the diversity of the microbial community was also reduced in the patient group (68). The identification of various dominant microorganisms in CP/CPPS will be more conducive to the future development of microbiology in CP/CPPS and its application in treatment.

The gut microbe is also an important aspect of microbial research. Shoskes et al. showed that the diversity of the gut microbial community in patients with chronic pelvic pain syndrome was significantly lower than that in the control group, and the count of Prevotella in patients with CP/CPPS was significantly lower than that in the control group (69). In contrast, Konkol et al. found a significant increase in the gut microbial population but a decrease in the levels of Bacteroides homogenes, Lactobacilli and Lactobacilli in animal models with prostatitis (70). By sequencing amplified polymerase chain products, Mandar et al. found that patients with prostatitis had a higher diversity of species than healthy men, as well as a higher proportion of Proteobacteria and a lower proportion of Lactobacillus (71). Therefore gut microbiota can be considered as a biomarker of disease and as a target for future research and treatment.

Murphy et al. injected a specific strain of S. epidermidis from healthy men into mice with autoimmune prostatitis and found that a cell wall component of NPI (lipoteichoic acid) mediated CTLA4-like ligand on prostate antigen presenting cells, resulting in increased expression of PDL1 and PDL2, decreasing the pain response to pelvic tactile abnormalities (72).

Kogan’s latest study categorized chronic bacterial prostatitis into aerobes dominant, anaerobes dominant, and both aerobes and anaerobes dominant groups, and found that more severe clinical conditions were observed in patients with both aerobes and anaerobes and titers ≥103 CFU/ml. Relate aerobic - anaerobic conditions to the degree of clinical status (73).

#### 4.1.2 Sexually transmitted pathogens

The occurrence of prostatitis is partly caused by the infection of sexually transmitted pathogens, such as Trichomonas vaginalis, Mycoplasma, chlamydia, etc. Early studies by Skerk et al. detected Chlamydia trachomatis, Trichomonas vaginalis, and Ureaplasma urealyticumfrom from prostatic secretions or in urine samples collected after prostatic massage (74). Though the detection rate of Chlamydia trachomatis in subsequent studies is not high, we still believe that when considering the possibility of chronic prostattis, we should carry out routine detection of Chlamydia trachomatis also should be carried out to confirm the pathogen (75). Proof may be that IgA of Chlamydia trachomatis is closely related to sperm concentration and normal morphology, Chlamydia trachomatis can destroy germ cells through immune mediation and reduce male fertility (76). Ureaplasma microtium, Ureaplasma urealyticum, and Mycoplasma gendii are commonly detected in type IIa chronic prostatitis, and recent studies have also found that Ureaplasma urealyticum plays a role in promoting calcification formation and high white sperm count in chronic prostate infection (77). Jang et al. demonstrated that infection with Trichomonas vaginitis in rats can lead to prostatitis, mainly manifested as pathological changes, infiltration of mast cells and increased production of the chemokine CCL2 (78).

#### 4.1.3 Fungi

For prostatitis in HIV patients, except bacteria and viruses, the most common infection is fungal pathogens, such as Candida, Cryptococcus, Aspergillus, Blastobacter, Coccidioides and Histoplasmosis, which can lead to disseminated infection and prostatitis in immunocompromised men (79).

### 4.2 The immune mechanism in the prostatitis microbiology

#### 4.2.1 Immune cells

Unlike the testis, the prostate is generally considered a non-immune organ, but many immune cells can be found in it, such as NK cells, mast cells, lymphocytes and macrophages. In the study conducted by Vykhovanets et al., it was first found that the prostate of normal healthy Sprague-Dawley rats contained an unusually high proportion of NK and NKT cells (80). Subsequent studies confirmed that the level of CD4(+) NKT cells decreased significantly and CD45RC(+)CD49(+)αβTCR(+) T cells increased in elderly SPI and EPI patients compared with elderly NPI and young patients (81). Therefore, identification of the phenotype of NK cells and NKT cells and their proportional relationship with T cells will be helpful for the diagnosis and treatment of chronic prostatitis. NK and NKT cells can also kill cancer cells. Recently, more research has been conducted on the relationship between prostate cancer and NK cells, and how to use NK cells for the treatment of prostate cancer. CP/CPPS are mainly characterized by their pelvic pain, and studies by Done et al. and Roman et al. show that the release of mast cell mediators is a key factor in pain. It can be observed that there are degranulation products of mast cells in the prostate and urine of CP/CPPS patients, mainly the increase of tryptase-β and carboxypeptidase A3. The expression of the receptor PAR2 of tryptase is increased in patients, which regulates the phosphorylation of the kinase ERK1/2 and the influx of calcium ions through extracellular signaling, leading to the occurrence of pain. In addition, nerve growth factor also seems to play a role in the induction of pain, but there are no definitive results (82, 83). The study of the correlation of mast cells provides new ideas for treating CP/CPPS patients, which can reduce pain in patients by inhibiting the tryptase-PAR2 axis. However, the etiology of CP/CPPS is currently unclear, and most studies believe that autoimmunity is an important cause. In early studies of autoimmune prostatitis induced in non-obese diabetic (NOD) mice, PSBP (steroid-binding protein) was found to be an autoantigen recognized by the NOD immune system, and CD4+ T cells played an important role in EAP (84). In the mouse model of autoimmune prostatitis (EAP)established by Motrich et al., prostatitis and chronic pelvic pain were mainly induced by Th1-related immune responses after prostate autoantigen-induced autoimmunity, and the deficiency of Th1 or Th2 cytokines reduced or enhanced susceptibility to autoimmune prostatitis, respectively. The adaptive immune response mediated by Th1 and Th17 through the production of cytokines is equally significant in CP/CPPS (85).

#### 4.2.2 Cytokines and chemokines

Cytokine changes also play an important role in prostatitis. Common cytokines and their changes are shown in **Table 3**. In the study of Motrich et al., an increase of IFN-γ and IL-12 was observed in the prostate of autoimmune animals, with a decrease in IL-10. The mouse model lacking IFN-γ signaling transcription factor had no inflammatory response. IFN-γ can also stimulate IL-15 production in the prostate by paracrine, thereby inducing the proliferation of prostate T cells and participating in the inflammatory process of the prostate, which proves that IFN-γ is involved in the pathogenesis of CP/CPPS (89). IFN-γ and IF-17A expression were found to be increased in mice with chronic prostatitis (96). Through the detection of cytokines in CP/CPPS patients, Penna et al. found that IL-8 may be the most reliable and predictive marker for the diagnosis of prostatitis, and IL-8 was significantly increased in type IIIa patients. In addition, the level of IL-8 was positively correlated with the patient’s symptom score and prostate specific antigen level (97). In addition to the measurement of cytokines, Penna et al. found an increase of CCL1, CCL3 and CCL4, CCL17 and CCL22, CXCL8 in the seminal plasma in patients with CP/CPPS or benign prostate hyperplasia (97). Quick et al. focused on the CCL2 and CCL3 pathways associated with pelvic pain. The CCL2-CCR2 axis and CCL3 are important mediators of pelvic pain, and only anti-CCL2 antibody is effective in the treatment of autoimmune prostatitis (98).

**Table 3:** Cytokines and prostatitis.

Cytokine	Species	Group	Sample Type	Regulation	Reference
CXCL-10	Mice	EAP&Normal	Prostate Tissue	Up	(86)
IL-1α	Rats	EAP&Normal	Prostate Tissue	Up	(87)
IL-1β	Rats	EAP&Normal	Prostate Tissue	Up	(87)
TNF-α	Rats	EAP&Normal	Prostate Tissue	Up	(87)
IL-4	Rats	EAP&Normal	Prostate Tissue	Up	(87)
IL-13	Rats	EAP&Normal	Prostate Tissue	Up	(87)
IL-17	Mice	EAP&Normal	Prostate Tissue	Up	(88)
IL-12	Mice	EAP&Normal	Prostate Tissue	Up	(89)
IL-10	Homo Sapiens	Chronic Bacterial Prostatitis infertile patients&Normal	Seminal Fluid	Down	(90)
IL-6	Homo Sapiens	Chronic Bacterial Prostatitis patients&Normal	Seminal Fluid	Up	(91)
IL-17	Rats	EAP&Normal	Prostate Tissue	Up	(92)
IL-17	Homo Sapiens	Chronic Bacterial Prostatitis patients&Normal	Seminal Fluid	Up	(93)
IFN-γ	Homo Sapiens	Chronic Bacterial Prostatitis patients&Normal	Seminal Fluid	Up	(93)
IL-1β	Mice	EAP&Normal	Blood	Up	(94)
IL-8	Homo Sapiens	Chronic Bacterial Prostatitis patients&Normal	Blood	Up	(95)
IL-2	Homo Sapiens	Chronic Bacterial Prostatitis patients&Normal	Blood	Up	(95)
IL-6	Homo Sapiens	Chronic Bacterial Prostatitis patients&Normal	Blood	Up	(95)
TNF-α	Homo Sapiens	Chronic Bacterial Prostatitis patients&Normal	Blood	Up	(95)

EAP, Experimental Autoimmune Prostatitis; CBPP, Chronic Bacterial Prostatitis patients; Up, Up-regulation of cytokine levels; Down, Down-regulation of cytokine levels.

#### 4.2.3 Inflammasome

In the process of activation of inflammatory innate immune system, in addition to cytokines playing an important role in the inflammatory process, inflammasome is also indispensable in promoting the formation and transformation of cytokines. Chronic bacterial prostatitis is through the upregulation of inflammasomes NLRP1 and NLRP3, and the increased expression of ASC and caspase-1, which together promote the conversion of downstream IL-1β and IL-18 precursors, promote the release of mature cytokines, and participate in the inflammatory and immune processes (99–101). Therefore, considering the role of inflammasome can provide a new target for the treatment of prostatitis.

#### 4.2.4 Biofilm

The generation of chronic bacterial prostatitis is associated with a variety of bacterial infections. These bacteria escape the immune by forming biofilms and have high tolerance to antibiotics, which affects the treatment of bacterial prostatitis. Kanamaru et al. showed that the possibility of biofilm formation from patients with prostatitis was higher than that of acute cystitis and pyelonephritis, and the isolates from prostatitis had higher optical density values and more curli fimbriae, which further confirmed the connection between biofilm and prostatitis (102).In addition, the hemolysin produced by E. coli cooperates with the biofilm to lead to the persistence of E. coli in the prostate (103). In a recent study, it was found that patients with biofilm formation had higher NIH-CPSI scores and less improvement in symptoms than patients without biofilm formation, which had a negative impact on antibiotic treatment (104).

The types of pathogens infected by different types of prostatitis will be different, so the identification of dominant microorganisms can be used as biomarkers to provide new targets and ideas for future disease research and treatment. The study of abnormal proportions of NK and NKT cells in patients with prostatitis, and their association with T cells, will also contribute to the treatment of chronic prostatitis and prostate cancer. The formation of cytokines and inflammasome is involved in the inflammatory and immune processes of prostate. The formation of cytokines promotes the assembly of inflammasome complex, and the appearance of inflammasome further promotes the release of mature cytokines, promoting the development of prostatitis. In the treatment of prostatitis, bacterial biofilm formation can affect the role of antibiotics and immune system, so how to improve the treatment of patients with prostatitis accompanied by biofilm is also very important.

## 5 Oligozoospermia, asthenospermia, azoospermia

### 5.1 Oligozoospermia, asthenospermia, azoospermia and microbiology

#### 5.1.1 Bacteria

Clinically, unilateral and bilateral infections of the testis and epididymis often lead to azoospermia and severe oligozoospermia, while gonadal infections are mostly caused by bacterial infections. Yang et al. revealed that the microbiomes of asthenospermia and oligozoospermia were significantly different in terms of β diversity, with high relative abundances of Bacteroides, Anaerobic Cocci, Spermicococcus, Lactobacillus and Acinetobacter Roche seen in asthenospermia patients, and significantly higher relative abundances of Lactobacillus in oligozoospermia patients (105). However, other studies have linked oligoasthenozoospermia in part to an increase in Neisseria, Klebsiella, and Pseudomonas pathogens and a decrease in Lactobacillus probiotics, so whether the effects of Lactobacillus on male fertility are beneficial or harmful remains controversial (105). Mehta et al. studied the effects of Streptococcus faecalis on oligozoospermia and teratozoospermia in men. Compared with semen containing micrococcus or α-Streptococcus hemolyticus and normal uninfected semen, the incidence of oligozoospermia and teratozoospermia was significantly higher, and the mean sperm concentration and the mean percentage of normal spermatozoa in semen were significantly lower (106).

#### 5.1.2 Sexually transmitted pathogens

Human papillomavirus and herpesviridae frequently infect epithelial cells and are considered to be considerable risk factors for infertility. In an earlier study conducted by Lai et al., asthenospermia was found to be significantly more common in HPV-infected patients (75%) than in men without HPV infection (8%) (107). Nasseri et al. looked at HPV detection rates in patients with oligozoospermia and azoospermia from another perspective and found significant differences in sperm counts and sperm motility rates between positive and negative samples from patients with oligozoospermia. In addition, HPV16, 45 genotypes in the high-risk group and HPV6, 11, 42 genotypes in the low-risk group were more common (108). HSV was found in 279 semen samples collected by Kurscheidt et al., HSV-1 was significantly associated with a lower mean sperm count, and HSV-2 infection was significantly associated with hemospermia and lower mean semen quality (109).

Ureurealyticum can damage sperm structure and function by reducing motility and causing damage to the sperm membrane, especially the lipid bilayer. In the analysis of various types and subgroups of Ureaplasma, Yang et al. found that subgroups A and C of Ureaplasma microgenesis and subgroup 1 of Ureaplasma urealyticum were associated with oligozoospermia, and subgroup 2 of Ureaplasma urealyticum may have the ability to impair semen motility (110).

Chlamydia trachomatis is also a common sexually transmitted pathogen, previous studies have found reduced sperm concentration, normal sperm morphology, sperm volume, sperm motility, and sperm velocity in chlamydia-positive samples compared with negative samples, and a 14-fold risk of reduced semen volume when both couples were tested for Chlamydia trachomatis (111).

#### 5.1.3 Other viruses

COVID-19 not only affects the respiratory system, but also has a certain impact on the reproductive function of patients. Thinning of spermatogenic tubules, interstitial edema, hyperemia, infiltration of inflammatory cells and inflammatory mediators can be observed in necropsy testis and epididymis specimens of patients with COVID-19. Compared with normal men, IL-6, TNF-α and MCP-1 in semen are increased, leading to low testosterone level and affecting normal spermatogenic function. 39.1% of the patients had oligozoospermia (39). The same phenomenon was observed in the study by Apaydin et al (112). In addition, after acute Zika virus infection, although there may be only early semen changes, which will return to normal values later, early pathological changes may also affect sperm function through testicular and epididymal pathological changes, thus affecting male fertility (113). Adeno-associated virus AAV was significantly higher in both oligoasthenospermia and oligozoospermia patients than in normal semen, but not in asthenospermia and azoospermia. AAV may cause oligoasthenospermia by infecting testes and interfering with normal sperm development (114).

### 5.2 The immune mechanism in the oligozoospermia, asthenospermia and azoospermia microbiology

#### 5.2.1 Immune cells and cytokines

In an earlier study of patients with azoospermia carried out by Mahmoud et al., it was observed that although T cells, B cells, macrophages and mast cells were all increased, only the increase in mast cells was significant. Due to the increase of tryptase, the number of mast cells were increased resulting in the testicles collagen fibers deposition obviously and affecting sperm production function (115). At the same time, the bacteria infection of the reproductive system may lead to leukopenia, which increases the number of anti-sperm antibodies by enhancing the function of T helper cells and B cells, and damages male fertility together with natural killer cells (116). In addition to the immune cell changes described above, proinflammatory factors are also changed in infertile men, especially in infertile men with genital tract infections. A recent study showed that for all cytokines measured in oligospermia, asthenospermia, and oligoasthenospermia compared with controls, only IL-5 levels were reduced, with significant reductions of 87%, 78%, and 87%, respectively (117).

#### 5.2.2 Antibody and immune complexes

In a meta-analysis of male infertility, ASA antibody positive patients had significantly lower sperm concentration and sperm motility than ASA negative controls (118). In addition to impairing sperm motility by activating the complement system, Dimitrov et al. also found that 15 out of 28 infertile men with asthenospermia were positive for anti-sperm CMI (cell-mediated immunity), thus ASA-mediated cellular immunity may be an important cause of oligozoospermia and asthenospermia (119). The immune complex also affects the male reproductive function. Murakami et al. found 4 disease-specific antigens in oligozoospermia patients and 5 disease-specific antigens in asthenospermia patients. The formation and deposition of immune complexes stimulate inflammation through the action of the complement system, leading to reproductive organ fibrosis and loss of related protein function, thus leading to spermatogenic dysfunction (120).

#### 5.2.3 HLA

Genes in HLA loci that play key roles in antigen presentation and immune response are strongly associated with asthenospermia and oligozoospermia. HLA-A*11:01 is associated with multiple forms of male infertility; HLA-DQB1*03:02 and HLA-A*29:02 are associated with oligozoospermia, and HLA-A*29:02 can also interfere with sperm count with HPV, as well as risk genes associated with oligozoospermia, they are HLA-DQA1*05:01, HLA-C*03:03, and HLA-DQB1*03:01.HLA was associated with male infertility and HPV to further explore the multiple influencing factors of male infertility (121).

The β diversity of microflora in oligospermia patients was significant, and the infection of micrococcus or α-hemolytic streptococcus was more (105, 106). The infection of sexually transmitted pathogens and viruses also participated in the process of sperm damage. Abnormal levels of IL-5 and specific gene locus changes of HLA can be used as biomarkers for infertile men (117, 121).

## 6 Conclusion

**Figure 1:**
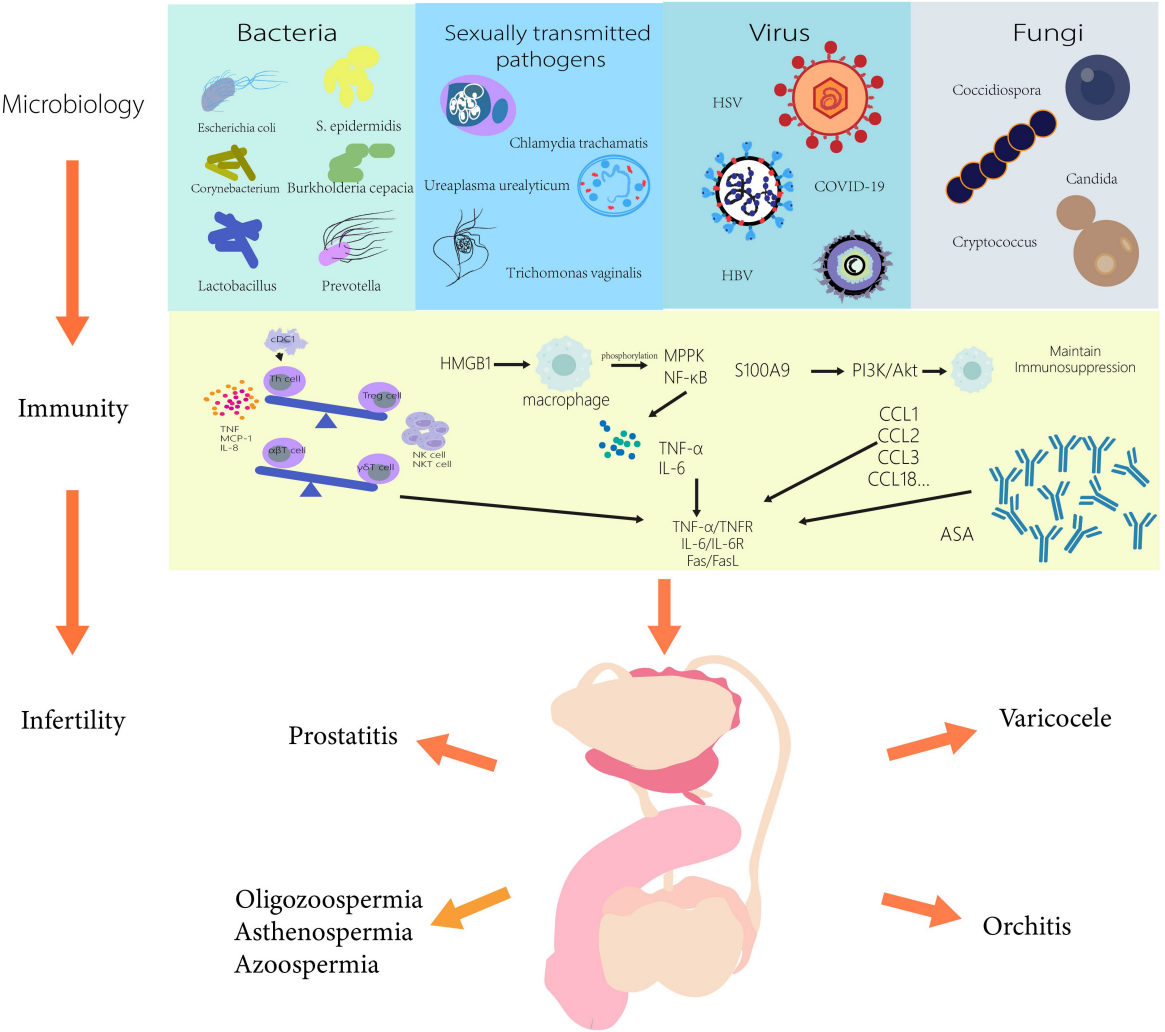
Microbiology and immune mechanisms associated with male infertility.

There is growing evidence linking male infertility to the microbiome, and rapidly evolving microbial sequencing and analysis methods will help us understand more potential pathogens associated with male infertility in the future. In this review, we summarize the current microorganisms affecting male fertility and summarize their immunological mechanisms, providing certain ideas for the subsequent treatment of male infertility in **Figure 1**. There are still some difficulties that we have not yet overcome. Obtaining the microorganisms in the research site without being affected is one of the most complex difficulties. For example, the presence of pathogens in semen analysis is extremely easy to be affected by the surrounding structure and is not completely targeted. Therefore, the methods of microbial sampling from different parts need to be further studied. In addition, by linking male infertility with the microbiome and exploring the immune mechanism in the microbiome, we can not only better understand the immune response *in vivo* under different pathological conditions, including the changes in the number and types of immune cells, immune factors, and chemokines, etc., but also provide more targeted immune target therapies for pathological conditions. It is also possible to combine immunotherapy with microbiomes therapy, which may be more effective in treating male infertility.

## Author contributions

JC prepared and wrote the manuscript; JYC oversaw the section, designed and finished the figure and tables; YF and QS commented on previous versions of the manuscript; KZ and CL revised the manuscript; HZ designed and revised the manuscript. All authors discussed the relevant literature and composed the sections of the review article. All authors contributed to the article and approved the submitted version.
